# The Temporal Version of the Pediatric Sepsis Biomarker Risk Model

**DOI:** 10.1371/journal.pone.0092121

**Published:** 2014-03-13

**Authors:** Hector R. Wong, Scott L. Weiss, John S. Giuliano, Mark S. Wainwright, Natalie Z. Cvijanovich, Neal J. Thomas, Geoffrey L. Allen, Nick Anas, Michael T. Bigham, Mark Hall, Robert J. Freishtat, Anita Sen, Keith Meyer, Paul A. Checchia, Thomas P. Shanley, Jeffrey Nowak, Michael Quasney, Arun Chopra, Julie C. Fitzgerald, Rainer Gedeit, Sharon Banschbach, Eileen Beckman, Kelli Harmon, Patrick Lahni, Christopher J. Lindsell

**Affiliations:** 1 Division of Critical Care Medicine, Cincinnati Children’s Hospital Medical Center and Cincinnati Children’s Research Foundation, Cincinnati, Ohio, United States of America; 2 Department of Pediatrics, University of Cincinnati College of Medicine, Cincinnati, Ohio, United States of America; 3 The Children’s Hospital of Philadelphia, Philadelphia, Pennsylvania, United States of America; 4 Division of Critical Care Medicine, Department of Pediatrics, Yale University School of Medicine, New Haven, Connecticut, United States of America; 5 Ann and Robert H. Lurie Children’s Hospital of Chicago, Chicago, Illinois, United States of America; 6 Children’s Hospital and Research Center Oakland, Oakland, California, United States of America; 7 Penn State Hershey Children’s Hospital, Hershey, Pennsylvania, United States of America; 8 Children’s Mercy Hospital, Kansas City, Missouri, United States of America; 9 Children’s Hospital of Orange County, Orange, California, United States of America; 10 Akron Children’s Hospital, Akron, Ohio, United States of America; 11 Nationwide Children’s Hospital, Columbus, Ohio, United States of America; 12 Children’s National Medical Center, Washington, DC, United States of America; 13 Morgan Stanley Children’s Hospital, Columbia University Medical Center, New York, New York, United States of America; 14 Miami Children’s Hospital, Miami, Florida, United States of America; 15 Texas Children’s Hospital, Houston, Texas, United States of America; 16 CS Mott Children’s Hospital at the University of Michigan, Ann Arbor, Michigan, United States of America; 17 Children’s Hospital and Clinics of Minnesota, Minneapolis, Minnesota, United States of America; 18 St. Christopher’s Hospital for Children, Philadelphia, Pennsylvania, United States of America; 19 Children’s Hospital of Wisconsin, Milwaukee, Wisconsin, United States of America; 20 Department of Emergency Medicine, University of Cincinnati College of Medicine, Cincinnati, Ohio, United States of America; University of Florida College of Medicine, United States of America

## Abstract

**Background:**

PERSEVERE is a risk model for estimating mortality probability in pediatric septic shock, using five biomarkers measured within 24 hours of clinical presentation.

**Objective:**

Here, we derive and test a temporal version of PERSEVERE (tPERSEVERE) that considers biomarker values at the first and third day following presentation to estimate the probability of a “complicated course”, defined as persistence of ≥2 organ failures at seven days after meeting criteria for septic shock, or death within 28 days.

**Methods:**

Biomarkers were measured in the derivation cohort (n = 225) using serum samples obtained during days 1 and 3 of septic shock. Classification and Regression Tree (CART) analysis was used to derive a model to estimate the risk of a complicated course. The derived model was validated in the test cohort (n = 74), and subsequently updated using the combined derivation and test cohorts.

**Results:**

A complicated course occurred in 23% of the derivation cohort subjects. The derived model had a sensitivity for a complicated course of 90% (95% CI 78–96), specificity was 70% (62–77), positive predictive value was 47% (37–58), and negative predictive value was 96% (91–99). The area under the receiver operating characteristic curve was 0.85 (0.79–0.90). Similar test characteristics were observed in the test cohort. The updated model had a sensitivity of 91% (81–96), a specificity of 70% (64–76), a positive predictive value of 47% (39–56), and a negative predictive value of 96% (92–99).

**Conclusions:**

tPERSEVERE reasonably estimates the probability of a complicated course in children with septic shock. tPERSEVERE could potentially serve as an adjunct to physiological assessments for monitoring how risk for poor outcomes changes during early interventions in pediatric septic shock.

## Introduction

We previously derived, updated, and validated the pediatric sepsis biomarker risk model (PERSEVERE; PEdiatRic SEpsis biomarkEr Risk modEl) [1], [2]. PERSEVERE is based on a decision tree approach. Classification and regression tree (CART) methodology was used to estimate 28-day mortality probability for pediatric septic shock based on a panel of five biomarkers and age, with an area under the receiver operating characteristic curve of 0.88. The biomarkers that were used to derive PERSEVERE were selected objectively based on extensive genome-wide expression studies designed for the discovery of candidate stratification biomarkers [1]–[4]. Furthermore, the biomarkers were measured from serum samples obtained during the first 24 hours of presentation to the pediatric intensive care unit (PICU) with septic shock, which is a clinically relevant time period for assigning mortality risk in this heterogeneous population.

While the ability of PERSEVERE to assign a reliable mortality probability during the initial stages of septic shock has inherent utility at multiple levels, it fails to consider temporal changes in biomarker levels, and how these temporal changes may further inform the estimation of risk for poor outcome. This is important because the natural history of septic shock is intrinsically dynamic and subject to change in response to therapy [5]–[7]. Consequently, the risk for poor outcome also changes over time and it is biologically plausible that temporal changes in the PERSEVERE biomarkers may reflect this change.

In the current study we have derived a temporal version of PERSEVERE (tPERSEVERE), which incorporates biomarker measurements at two time points during the initial three days of illness to estimate the probability of a poor outcome termed “complicated course”. We subsequently test the prognostic accuracy of tPERSEVERE in an independent test cohort.

## Methods

### Ethics Statement

The Institutional Review Boards (IRB) of each participating institution approved secondary use of biological specimens and clinical data: Cincinnati Children’s Hospital Medical Center, The Children’s Hospital of Philadelphia, Yale University School of Medicine, Ann & Robert H. Lurie Children’s Hospital of Chicago, Children’s Hospital and Research Center Oakland, Penn State Hershey Children’s Hospital, Children’s Mercy Hospital, Children’s Hospital of Orange County, Akron Children’s Hospital, Nationwide Children’s Hospital, Children’s National Medical Center, Morgan Stanley Children’s Hospital, Columbia University Medical Center, Miami Children’s Hospital, Texas Children’s Hospital, CS Mott Children’s Hospital at the University of Michigan, St. Christopher’s Hospital for Children, and Children’s Hospital of Wisconsin. Written consent was obtained from the parents or legal guardians of all subjects enrolled, unless stated otherwise.

### Derivation Cohort Study Subjects

Seventeen institutions contributed biological specimens and clinical data to a central repository, with approval from the Institutional Review Boards of each participating institution. Data collection methods were previously described in detail [1], [8]. Briefly, children ≤10 years of age admitted to the PICU and meeting pediatric-specific criteria for septic shock were eligible for enrollment. After informed consent from parents or legal guardians, serum samples were obtained within 24 hours of initial presentation to the PICU with septic shock; these are referred to as “day 1” samples. Forty-eight hours after obtaining day 1 samples, a second serum sample was obtained if possible; these are referred to as “day 3” samples. Of the 355 subjects in the original PERSEVERE derivation and validation cohorts, there were 225 with biomarker data available for both day 1 and day 3. The current analysis included these 225 subjects, all of whom were enrolled between May 2002 and August 2010.

The 130 subjects excluded because day 3 biomarker data were not available were enrolled between May 2005 and December 2011. The mortality rate among the excluded subjects (19.2%) was significantly higher than that of the included subjects. Among the excluded subjects who died, 56% of the deaths occurred before day 3. The excluded and included subjects were otherwise not significantly different with respect to age, pediatric risk of mortality (PRISM) score, and incidence of a complicated course.

### Test Cohort Study Subjects

The test cohort subjects were pooled from four sources, with approval from the respective Institutional Review Boards. Thirty-three subjects were included from an ongoing genomics study in pediatric septic shock being conducted at 17 participating institutions [8]–[17]. The enrollment criteria are identical to those for the derivation cohort. The current analysis included subjects enrolled between September 2011 and May 2013, which represents subjects enrolled after the original derivation and validation of PERSEVERE.

Eleven subjects were included from among those enrolled in a prospective quality improvement program at one institution. This institution uses PERSEVERE to benchmark outcomes for all patients admitted to the PICU with septic shock. Enrollment procedures are identical to those described above, except that there is no age restriction and the Institutional Review Board of Cincinnati Children’s Hospital Medical Center granted permission for waiver of informed consent. Serum samples were collected from residual blood samples in the clinical laboratory. Subjects from this source were enrolled between September 2012 and May 2013.

Nineteen subjects (age range: 8 days to 18 years) were participants in a prospective, observational study at Ann & Robert H. Lurie Children’s Hospital of Chicago, Chicago, Illinois, evaluating nitric oxide metabolism and mitochondrial function in children with septic shock [18]. Of the 30 subjects with septic shock enrolled in that study, 19 had serum samples available for analysis. The current analysis included subjects enrolled between May 2009 and June 2010.

Eleven subjects (age range: 2 to 20 years old) were participants in a prospective, observational study at Yale-New Haven Children’s Hospital, New Haven, Connecticut, evaluating angiopoietin levels in children with septic shock [19]. Of the 17 subjects with septic shock enrolled in that study, 11 had serum samples available for analysis. The current analysis included subjects enrolled between September 2009 and December 2011.

### Study Procedures

For all studies, annotated clinical and laboratory data were collected daily while the participant was in the PICU. Illness severity was assessed at the time of presentation using the PRISM score [20]. The number of organ failures during the initial 7 days of PICU admission was recorded using pediatric-specific criteria [21]. All-cause mortality was tracked for 28 days after meeting criteria for septic shock. Our composite endpoint termed “complicated course”, is defined as persistence of two or more organ failures at seven days after meeting criteria for septic shock, or death within 28 days of presentation, as previously described [22]–[24].

### Biomarkers

PERSEVERE includes C-C chemokine ligand 3 (CCL3), interleukin 8 (IL8), heat shock protein 70 kDa 1B (HSPA1B), granzyme B (GZMB), and matrix metallopeptidase 8 (MMP8). Serum concentrations of these biomarkers were measured using a multi-plex magnetic bead platform (MILLIPLEX MAP) designed for this project by the EMD Millipore Corporation (Billerica, MA). Biomarker concentrations were measured in a Luminex 100/200 System (Luminex Corporation, Austin, TX), according the manufacturers’ specifications. Assay performance data were previously published [1].

### Statistical Analysis

Initially, data are described using medians, interquartile ranges, frequencies, and percentages. Comparisons between groups used the Mann-Whitney U-test, Chi-square, or Fisher’s Exact tests as appropriate. Descriptive statistics and comparisons used SigmaStat Software (Systat Software, Inc., San Jose, CA).

CART analysis was used to derive tPERSEVERE (Salford Predictive Modeler v7.0, Salford Systems, San Diego, CA) [1], [2], [25], [26]. The primary outcome variable for the modeling procedures is complicated course. The absolute day 1 and day 3 biomarker values, the percentage change in biomarker values from day 1 to day 3, and age were considered in the modeling procedures. Weighting of cases and the addition of cost for misclassification were not used in the modeling procedures. The code used to generate the model is available from the authors. Performance of the derived model is reported using diagnostic test statistics with 95% confidence intervals computed using the score method as implemented by the VassarStats Website for Statistical Computation [27].

## Results

### Derivation of tPERSEVERE

Table 1 shows the demographic and clinical characteristics of the derivation cohort (n = 225). The 52 (23%) subjects with a complicated course had a higher median PRISM score and were less likely to have a causative organism isolated compared to the 173 subjects with a non-complicated course. No other differences were observed.

**Table 1:** Demographics and clinical characteristics of the derivation and test cohorts (CC = complicated course).

	Derivation Cohort			Test Cohort		
	All	Non-CC	CC	All	Non-CC	CC
N	225	173	52	74	58	16
Mortality (%)	7	0	31	5	0	25
Median age years	2.3	2.4	1.5	5.7	5.7	5.8
(IQR)	(0.8–5.6)	(1.0–6.0)	(0.7–4.4)	(1.7–12.2)^3^	(1.7–12.2)	(1.1–14.1)
Median PRISM score	14	12	21	11	11	14
(IQR)^1^	(9–21)	(8–18)	(12–26)^2^	(9–19)	(7–19)	(11–20)
Males # (%)	141 (63)	105 (61)	36 (69)	37 (50)	31 (53)	6 (38)^3^
Females # (%)	84 (37)	68 (39)	16 (31)	37 (50)	27 (47)	10 (62)
Caucasian # (%)	160 (71)	126 (73)	34 (65)	50 (68)	38 (66)	12 (75)
African American # (%)	37 (16)	28 (16)	9 (17)	7 (9)	5 (9)	2 (13)
Other Race # (%)	13 (6)	9 (5)	4 (8)	1 (1)	1 (2)	0 (0)
Unreported Race # (%)	15 (7)	10 (6)	5 (10)	16 (22)^3^	14 (24)	2 (13)
Gram+bacteria # (%)	61 (27)	43 (25)	18 (35)	20 (27)	14 (24)	6 (38)
Gram - bacteria # (%)	64 (28)	45 (26)	19 (37)	14 (19)	10 (17)	4 (25)
Viral infection # (%)	23 (10)	15 (9)	8 (15)	3 (4)	3 (5)	0 (0)
Fungal infection # (%)	3 (1)	2 (1)	1 (2)	3 (4)	3 (5)	0 (0)
No organism # (%)	82 (36)	71 (41)	11 (21)^2^	37 (50)^3^	31 (53)	6 (38)
Any co-morbidity (%)	98 (44)	78 (45)	20 (38)	28 (38)	23 (40)	5 (31)
Malignancy # (%)	16 (7)	14 (8)	2 (4)	12 (16)^3^	9 (16)	3 (19)
Immunesuppression # (%)^4^	32 (14)	28 (16)	4 (8)	6 (8)	5 (9)	1 (6)

1 Nineteen subjects (15 with a non-complicated course and 4 with a complicated course) in the test cohort did not have available PRISM scores.

2 p<0.05 vs. derivation cohort subjects with a non-complicated course.

3 p<0.05 vs. derivation cohort.

4 Refers to patients with immune suppression not related to cancer (for example, those receiving immune suppressive medication for solid organ or bone marrow transplantation, or those with a primary immune deficiency).

**Figure 1:**
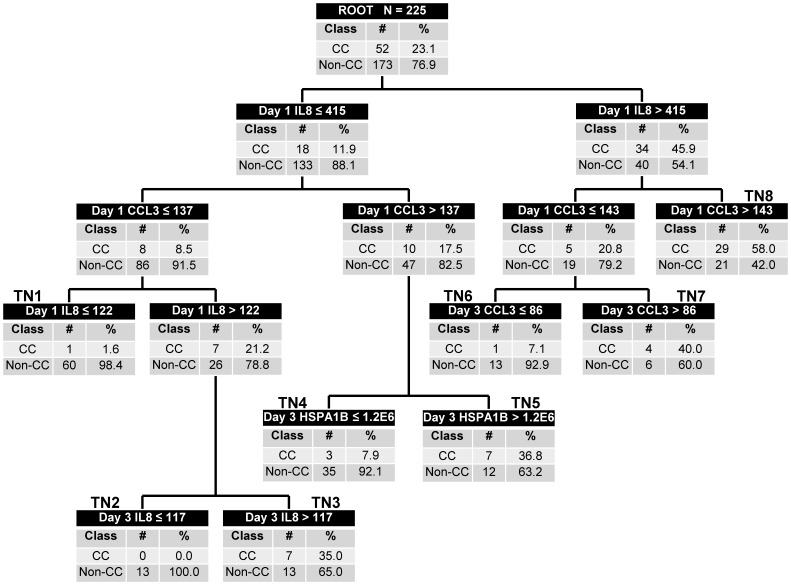
Classification tree from the derivation cohort (N = 225). The classification tree consists of 7 biomarker-based decision rules and 14 daughter nodes. The classification tree includes day 1 and day 3 data for interleukin-8 (IL8) and C-C chemokine ligand 3 (CCL3), and day 3 data heat shock protein 70 kDa 1B (HSPA1B). Each node provides the biomarker serum concentration-based decision rule, and the number of subjects with and without complicated course (CC), with the respective rates. For consistency, the serum concentrations of all biomarkers are provided in pg/ml. Terminal nodes (TN) 1, 2, 4, and 6 are considered low risk nodes, whereas terminal nodes 3, 5, 7, and 8 are considered high-risk terminal nodes. To calculate the diagnostic test characteristics, all subjects in the low risk terminal nodes (n = 126) were classified as predicted to not have a complicated course, whereas all subjects in the high risk terminal nodes (n = 99) were classified as predicted to have a complicated course.

Figure 1 shows the derived model. Maximum accuracy was achieved with five biomarker variables: absolute day 1 IL8 and CCL3 values; and absolute day 3 IL8, CCL3, and HSPA1B values. None of the other biomarker variables or age contributed to predictive accuracy. There were four low probability terminal nodes for a complicated course (0.0 to 7.9% probability; terminal nodes TN1, TN2, TN4, and TN6) and four high probability terminal nodes (35 to 58% probability; TN3, TN5, TN7, and TN8). Among the 126 subjects classified as low probability, 121 (96%) had a non-complicated course and five (4%) had a complicated course. Among the 99 subjects classified as high probability, 47 (47%) had a complicated course. Table 2 shows the diagnostic test characteristics of the derived decision tree.

**Table 2:** Test characteristics of the decision tree.

	Derivation Cohort	Test Cohort	Updated Model
Number of Subjects	225	74	299
Number of True Positives	47	13	62
Number of True Negatives	121	47	162
Number of False Positives	52	11	69
Number of False Negatives	5	3	6
Sensitivity	90% (78–96)	81% (54–95)	91% (81–96)
Specificity	70% (62–77)	81% (68–90)	70% (64–76)
Positive Predictive Value	47% (37–58)	54% (33–74)	47% (39–56)
Negative Predictive Value	96% (91–99)	94% (82–98)	96% (92–99)
+Likelihood Ratio	3.0(2.4–3.8)	4.3 (2.4–7.7)	3.1 (2.5–3.8)
−Likelihood Ratio	0.1 (0.1–0.3)	0.2 (0.1–0.6)	0.1 (0.1–0.3)
Area Under the Curve	0.85 (0.79–0.90)	0.83 (0.74–0.93)	0.84 (0.79–0.89)

### Testing tPERSEVERE

The independent test cohort consisted of 74 subjects with septic shock, of whom 16 (22%) had a complicated course. Table 1 shows the demographics and clinical characteristics of the test cohort. Compared to the derivation cohort, the test cohort subjects had a higher median age, and a higher proportion had no race reported, no causative organism isolated, and malignancy. Within the test cohort, the subjects with a complicated course had a lower proportion of males, compared to the subjects with a non-complicated course. No other differences were observed.

The test cohort subjects were classified according to the derived model. Among the 50 subjects classified as low probability for a complicated course, 47 (94%) had a non-complicated course and three (6%) had a complicated course. Among the 24 subjects classified as high probability, 13 (54%) had a complicated course. Table 2 shows the diagnostic test characteristics of tPERSEVERE in the test cohort.

### Updating tPERSEVERE

**Figure 2:**
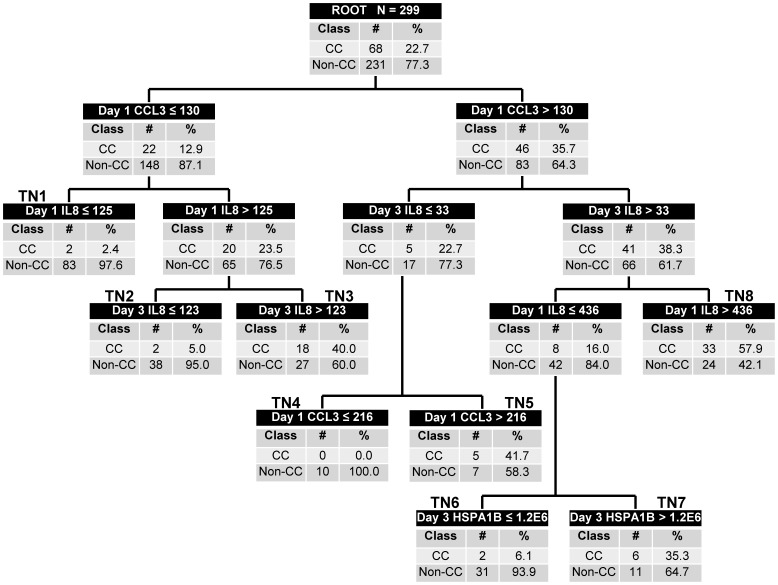
Classification tree from the updated model based on the combined derivation and test cohorts (N = 299). The classification tree consists of 7 biomarker-based decision rules and 14 daughter nodes. The classification tree includes day 1 and 3 interleukin-8 (IL8 data), day 1 C-C chemokine ligand 3 (CCL3) data, and day 3 heat shock protein 70 kDa 1B (HSPA1B) data. Each node provides the biomarker serum concentration-based decision rule, and the number of subjects with and without a complicated course (CC), with the respective rates. For consistency, the serum concentrations of all stratification biomarkers are provided in pg/ml. Terminal nodes (TN) 1, 2, 4, and 6 are considered low risk nodes for a complicated course, whereas terminal nodes 3, 5, 7, and 8 are considered high-risk terminal nodes for a complicated course. To calculate the diagnostic test characteristics, all subjects in the low risk terminal nodes (n = 168) were classified as predicted to not have a complicated course, whereas all subjects in the high risk terminal nodes (n = 131) were classified as predicted to have a complicated course.

tPERSEVERE was updated using all 299 subjects in the combined derivation and test cohorts. We again considered all potential biomarker variables and age in the updating process. Figure 2 shows the updated version of tPERSEVERE. Maximum accuracy was achieved with the same biomarker variables as the originally derived decision tree, except that day 3 CCL3 data no longer added to the predictive accuracy. In addition, a day 1 CCL3-based decision rule replaced the day 1 IL8-based, first-level decision rule in the originally derived decision tree. The updated version of tPERSEVERE contains four low probability terminal nodes for a complicated course (0.0 to 6.1% probability; TN1, TN2, TN4, and TN6), and four high probability terminal nodes (35.3 to 57.9% probability; TN3, TN5, TN7, and TN8). Among the 168 subjects classified as low probability, 162 (96%) had a non-complicated course and six (4%) had a complicated course. Among the 131 subjects classified as high probability, 62 (47%) had a complicated course. Table 2 shows the diagnostic test characteristics of the updated version of tPERSEVERE.

## Discussion

The temporal version of PERSEVERE (tPERSEVERE) reasonably estimates the risk of a complicated course in a heterogeneous cohort of children with septic shock. The study subjects were drawn from multiple centers and pooled from four distinct databanks, thus adding substantial variability with regard to pathology and therapeutic interventions. Despite the concern that such heterogeneity might diminish the accuracy of predictions, tPERSEVERE performed reliably. This suggests that tPERSEVERE will be generalizable upon further testing.

We note that the positive predictive value of tPERSEVERE is substantially lower than the negative predictive value. The positive and negative predictive values of a diagnostic test are influenced by the prevalence of the outcome of interest [3]. In this study, the prevalence of a complicated course was about 23%, so one would expect that the positive predictive value would be lower than the negative predictive value. Further, if one assumes that therapeutic interventions are beneficial and can ameliorate the risk of a poor outcome, then some of the false positives (which lower the positive predictive value and specificity) likely represent patients in whom the predicted poor outcome was prevented by therapeutic interventions.

The high sensitivity allows one to reliably identify patients at risk for a poor outcome, while the high negative predictive value allows one to identify those who are low risk. Indeed, a dichotomous interpretation of the model is that it can be used to divide a heterogeneous cohort of children with septic shock into two groups that differ by a factor of ten in the probability of a poor outcome. An alternative interpretation of the model is to view each terminal node individually, which allows for the assignment of a range of probabilities for a complicated course.

In the current study, we focused our modeling procedures on a composite outcome variable, complicated course, whereas in our previous studies we focused on 28-day mortality [1], [2]. There are two primary reasons for this change in focus. First, while 28-day mortality is an important outcome variable, mortality alone does not fully capture all septic shock-associated morbidity. Organ failure has been associated with poor functional outcomes in septic shock survivors [28], and so the composite variable used in this study has been recently proposed as a clinically relevant study endpoint [22], [23]. Second, the incidence of mortality in the study cohorts was too low for reliable modeling. We note that the five false negative subjects in the derivation cohort and the three false negatives in the test cohort all survived. This suggests that tPERSEVERE has high reliability for predicting mortality, even though it was derived to estimate the risk of a complicated course.

We previously compared the performance of PERSEVERE to that of PRISM, and found that PERSEVERE outperformed PRISM [1], [2]. We have not compared tPERSEVERE to PRISM because the latter is not intended to be used as a temporal scoring system. We propose that tPERSEVERE could be used as an adjunct to traditional physiological parameters for monitoring therapeutic interventions in children with septic shock. Assuming that the risk of a complicated course is modified by therapy, tPERSEVERE provides an objective readout of therapeutic effectiveness by comparison to the baseline risk predicted by PERSEVERE. A changing risk, reflected by changing biomarkers, might even serve as a surrogate outcome variable in Phase 1 or 2 interventional clinical trials.

We note that in the initially derived tPERSEVERE, 49% of the derivation cohort subjects and 47% of the test cohort subjects occupy terminal nodes 1 and 8, which are dependent only on day 1 data. However, in the updated model, there is only one terminal node that is dependent exclusively on day 1 data (TN1), and only 28% of the subjects occupy this node. The remaining terminal nodes are informed by both day 1 and day 3 biomarker data. This suggests that as we accrue more data pertaining to changing biomarkers, we may continue to improve our ability to both predict risk and monitor therapeutic effect.

We note the limitations of our study. The test cohort, while representative of the general pediatric septic shock population, is relatively small. There is potential for selection bias in the derivation cohort because not all of the original PERSEVERE subjects were available for deriving tPERSEVERE. tPERSEVERE requires prospective testing; we are currently in the process of enrolling this prospective test cohort. Finally, tPERSEVERE, in the absence of PERSEVERE, does not capture early septic shock mortality (i.e. <3 days).

In conclusion, we have derived, tested, and updated a temporal version of PERSEVERE. Pending further prospective validation, we propose that tPERSEVERE has potential to serve as an adjunct to physiological assessments for monitoring how risk for poor outcomes changes during the period of early intervention in children with septic shock, or to serve as a surrogate outcome variable in clinical trials.

## References

[1] Wong HR, Salisbury S, Xiao Q, Cvijanovich NZ, Hall M, et al (2012) The pediatric sepsis biomarker risk model. Crit Care 16: R174. 23025259 10.1186/cc11652 PMC3682273

[2] Wong HR, Weiss SL, Giuliano JS, Jr., Wainwright MS, Cvijanovich NZ, et al.. (2014) Testing the prognostic accuracy of the updated pediatric sepsis biomarker risk model. PLoS One (in press, DOI 10.1371/journal.pone.0086242). 10.1371/journal.pone.0086242 PMC3906040 24489704

[3] Kaplan JM, Wong HR (2011) Biomarker discovery and development in pediatric critical care medicine. Pediatr Crit Care Med 12: 165–173. 20473243 10.1097/PCC.0b013e3181e28876 PMC2924462

[4] Wong HR, Lindsell CJ, Pettila V, Meyer NJ, Thair SA, et al.. (2013) A Multibiomarker-Based Outcome Risk Stratification Model for Adult Septic Shock. Crit Care Med. 10.1097/CCM.0000000000000106 PMC4620515 24335447

[5] Hanna W, Wong HR (2013) Pediatric sepsis: challenges and adjunctive therapies. Crit Care Clin 29: 203–222. 23537672 10.1016/j.ccc.2012.11.003 PMC3612267

[6] Wong HR (2013) Genome-wide expression profiling in pediatric septic shock. Pediatr Res 73: 564–569. 23329198 10.1038/pr.2013.11 PMC3615026

[7] Wynn J, Cornell TT, Wong HR, Shanley TP, Wheeler DS (2010) The host response to sepsis and developmental impact. Pediatrics 125: 1031–1041. 20421258 10.1542/peds.2009-3301 PMC2894560

[8] Wong HR, Shanley TP, Sakthivel B, Cvijanovich N, Lin R, et al (2007) Genome level expression profiles in pediatric septic shock indicate a role for altered zinc homeostasis in poor outcome. Physiol Genomics 30: 146–155. 17374846 10.1152/physiolgenomics.00024.2007 PMC2770262

[9] Cvijanovich N, Shanley TP, Lin R, Allen GL, Thomas NJ, et al (2008) Validating the genomic signature of pediatric septic shock. Physiol Genomics 34: 127–134. 18460642 10.1152/physiolgenomics.00025.2008 PMC2440641

[10] Shanley TP, Cvijanovich N, Lin R, Allen GL, Thomas NJ, et al (2007) Genome-level longitudinal expression of signaling pathways and gene networks in pediatric septic shock. Mol Med 13: 495–508. 17932561 10.2119/2007-00065.Shanley PMC2014731

[11] Wong HR, Cvijanovich N, Allen GL, Lin R, Anas N, et al (2009) Genomic expression profiling across the pediatric systemic inflammatory response syndrome, sepsis, and septic shock spectrum. Crit Care Med 37: 1558–1566. 19325468 10.1097/CCM.0b013e31819fcc08 PMC2747356

[12] Wong HR, Cvijanovich N, Lin R, Allen GL, Thomas NJ, et al (2009) Identification of pediatric septic shock subclasses based on genome-wide expression profiling. BMC Med 7: 34. 19624809 10.1186/1741-7015-7-34 PMC2720987

[13] Wynn JL, Cvijanovich NZ, Allen GL, Thomas NJ, Freishtat RJ, et al (2011) The influence of developmental age on the early transcriptomic response of children with septic shock. Mol Med 17: 1146–1156. 21738952 10.2119/molmed.2011.00169 PMC3321808

[14] Basu RK, Standage SW, Cvijanovich NZ, Allen GL, Thomas NJ, et al (2011) Identification of candidate serum biomarkers for severe septic shock-associated kidney injury via microarray. Crit Care 15: R273. 22098946 10.1186/cc10554 PMC3388679

[15] Wong HR, Cvijanovich NZ, Allen GL, Thomas NJ, Freishtat RJ, et al (2011) Validation of a gene expression-based subclassification strategy for pediatric septic shock. Crit Care Med 39: 2511–2517. 21705885 10.1097/CCM.0b013e3182257675 PMC3196776

[16] Wong HR, Freishtat RJ, Monaco M, Odoms K, Shanley TP (2010) Leukocyte subset-derived genomewide expression profiles in pediatric septic shock. Pediatr Crit Care Med 11: 349–355. 20009785 10.1097/PCC.0b013e3181c519b4 PMC2927840

[17] Wong HR, Cvijanovich N, Wheeler DS, Bigham MT, Monaco M, et al (2008) Interleukin-8 as a stratification tool for interventional trials involving pediatric septic shock. Am J Respir Crit Care Med 178: 276–282. 18511707 10.1164/rccm.200801-131OC PMC2542425

[18] Weiss SL, Haymond S, Ralay Ranaivo H, Wang D, De Jesus VR, et al (2012) Evaluation of asymmetric dimethylarginine, arginine, and carnitine metabolism in pediatric sepsis. Pediatr Crit Care Med 13: e210–218. 22460770 10.1097/PCC.0b013e318238b5cd PMC3392424

[19] Giuliano JS, Jr., Tran K, Li FY, Northrup V, Tala JA, et al.. (2013) The temporal kinetics of circulating angiopoietin levels in children with sepsis. Pediatr Crit Care Med. 10.1097/PCC.0b013e3182a553bb PMC3947338 24141659

[20] Pollack MM, Patel KM, Ruttimann UE (1997) The Pediatric Risk of Mortality III–Acute Physiology Score (PRISM III-APS): a method of assessing physiologic instability for pediatric intensive care unit patients. J Pediatr 131: 575–581. 9386662 10.1016/s0022-3476(97)70065-9

[21] Goldstein B, Giroir B, Randolph A (2005) International pediatric sepsis consensus conference: definitions for sepsis and organ dysfunction in pediatrics. Pediatr Crit Care Med 6: 2–8. 15636651 10.1097/01.PCC.0000149131.72248.E6

[22] Mickiewicz B, Vogel HJ, Wong HR, Winston BW (2013) Metabolomics as a novel approach for early diagnosis of pediatric septic shock and its mortality. Am J Respir Crit Care Med 187: 967–976. 23471468 10.1164/rccm.201209-1726OC PMC3707368

[23] Abulebda A, Cvijanovich N, Thomas NJ, Allen GL, Anas N, et al (2013) Post-intensive care unit admission fluid balance and pediatric septic shock outcomes: A risk-stratified analysis. Crit Care Med 42: 397–403. 10.1097/CCM.0b013e3182a64607 PMC3947064 24145842

[24] Xiao W, Mindrinos MN, Seok J, Cuschieri J, Cuenca AG, et al (2011) A genomic storm in critically injured humans. J Exp Med 208: 2581–2590. 22110166 10.1084/jem.20111354 PMC3244029

[25] Che D, Liu Q, Rasheed K, Tao X (2011) Decision tree and ensemble learning algorithms with their applications in bioinformatics. Adv Exp Med Biol 696: 191–199. 21431559 10.1007/978-1-4419-7046-6_19

[26] Muller R, Mockel M (2008) Logistic regression and CART in the analysis of multimarker studies. Clin Chim Acta 394: 1–6. 18455512 10.1016/j.cca.2008.04.007

[27] VassarStats Website for Statistical Computation. http://faculty.vassar.edu/lowry/VassarStats.html. Accessed September 1, 2013.

[28] Typpo KV, Petersen NJ, Hallman DM, Markovitz BP, Mariscalco MM (2009) Day 1 multiple organ dysfunction syndrome is associated with poor functional outcome and mortality in the pediatric intensive care unit. Pediatr Crit Care Med 10: 562–570. 19741445 10.1097/PCC.0b013e3181a64be1 PMC2780005

